# The effect of a simulation-based training program in basic life support on the knowledge of Palestinian nurses: a quasi-experimental study in governmental hospitals

**DOI:** 10.1186/s12912-023-01552-x

**Published:** 2023-10-20

**Authors:** Yousef Fahajan, Osama J. Emad, Ahmed Hassan Albelbeisi, Ali Albelbeisi, Yasmin Abu Shnena, Ayda Khader, Edris Kakemam

**Affiliations:** 1 General Directorate of Nursing, Ministry of Health, Gaza, Palestine; 2 General Directorate of Mental Health, Ministry of Health, Gaza, Palestine; 3 Medical Services Directorate, Gaza Strip, Palestine; 4 College of Health Professions, Israa University, Gaza, Palestine; 5 Health Research Unit, Ministry of Health, Gaza, Palestine; 6 Faculty of Nursing, Midwifery Department, Islamic University of Gaza, Gaza, Palestine; 7 Clinical Research Development Unit of Tabriz Valiasr Hospital, Tabriz University of Medical Sciences, Tabriz, Iran

**Keywords:** Simulation, BLS Knowledge, BLS Practice, Nurses

## Abstract

**Background:**

Basic Life Support (BLS) plays an important role in increasing the survival rate of hospitalized heart attack patients. There are no previous studies on the effect of BLS training among Palestinian nurses. This study aimed to evaluate the effect of simulation-based BLS training program on nurses’ knowledge Palestinian nurses at governmental hospitals.

**Methods:**

A quasi-experimental, pre & post-test design was used. 700 nurses were recruited proportionally using a simple random sampling method among 2980 nurses from 13 public hospitals in the Gaza Strip. This study was conducted from June to August 2022. A practical BLS test consisting of 10 multiple-choice questions according to American Heart Association guidelines (2020) was collected and sociodemographic characteristics. SPSS software, version 24 was used for the statistical analysis. Descriptive statistics and weighted mean were used. T-Test and One-way analysis of variance (ANOVA) were applied to determine differences in means among groups.

**Results:**

Most of the participating nurses (55.7%) were male, while (44.3%) were female. The majority of nurses (84.4%) are under 40 years of age. The weighted mean scores in the pre-test ranged from 52.2 to 75.1% and the mean scores was (6.16 ± 1.97). After applying conventional BLS training, the weighted mean scores ranged from 85.6 to 97.3% and the mean scores was (9.19 ± 1.04). The study revealed that the nurses’ knowledge increased after applying simulation-based training program. The mean of knowledge scores was statistically significant between the pre and post-test on the basis of the current work hospital (P-value < 0.001).

**Conclusion:**

This study affords significant evidence of the positive effects of the BLS training program in improving nurses’ knowledge; we recommend advanced BLS training for all healthcare providers, doctors, and nurses working in hospitals and healthcare centers. Nursing managers can implement systematic strategies to enhance nurses’ knowledge and practice in BLS to target low-scoring Governorates.

## Background

A heart attack is an unexpected cessation of the functional activity of the heart due to failure of the heart to pump blood successfully [1]. Heart attack as a life-threatening state needs urgent interventions to ensure the lives of individuals and avoid irreversible damage to vital organs. According to the American Heart Association (AHA), cardiovascular disease has been identified as the number one cause of death, accounting for more than 17.9 million deaths per year in 2015, a number that is anticipated to extend to more than 23.6 million by 2030 [2]. A heart attack a major health problem that is estimated to be responsible for 15–20% of all deaths [3]. Several studies showed that the cardiac arrest survival rate decreases by 5–10% for each minute within the frame of cardiopulmonary resuscitation, which is an integral component of (BLS) [4]. BLS serves as a critical determinant of patient survival from heart attack. Training is vital in the enhancement of Cardiopulmonary Resuscitation (CPR) effects. BLS includes recognition, support of ventilation, and circulation in the episode of heart attack. It has a mixture of abilities including mouth-to-mouth breathing to support ventilation and chest compressions to normalize blood circulation to the brain and vital organs, called (CPR). BLS may be a basic and very effective method that allows anyone to help assist maintain life, soon after heart attack [5].

Knowledge and practice of nurses regarding BLS strategies ensure that the patient survives long sufficient [6].Nurses’ knowledge of BLS and proper practice of the techniques and maneuvers enable a person to effectively resuscitate a patient and its importance in saving lives [7].Simulation-based BLS training is a viable way of exchanging abilities with trainees in a cost-effective way. It provides an optimum way for employers to assess how well their trainees are putting abilities into practice, and the decisions they are making in front of simulated real-life circumstances [8]. Using simulation-based BLS training ultimately extends the quality of treatment and patient safety through a greater commitment by healthcare professionals to principles-based resuscitation approaches [9]. Also, the use of the simulation approach can increase the knowledge, skills, and performance of the nursing practice; they can reach high levels of critical thinking and obtain new professional skills without putting the patient’s health at risk [10]. A previous systematic review study aimed to evaluate effect of simulation-based training on nurses’ skills and knowledge, demonstrated that the simulation training appears to be an effective strategy for improving nurses’ knowledge and skills [11]. In addition, a qualitative study conducted among midwifery students in Sweden showed that simulation created links between theory and practice, and provided a safe learning environment [12].To the best of our knowledge, there are no previous studies about BLS training in Palestine. Therefore, this study aimed to evaluate the effect of simulation-based BLS training on nurses’ knowledge among nurses at governmental hospitals in the Gaza strip, Palestine.

## Methods

### Design and setting

A quasi-experimental design, pre& post-test conducted from June to August 2022 in 13 governmental hospitals located in the five Gaza Strip Governorates’. Gaza Strip is a small portion of the Occupied Palestinian Territories and is divided into five small governorates(Rafah, Khan-Younis, Middle-zone, Gaza, and North-Gaza) with an inhabitant of more than two million [13, 14]. The included hospitals have various services and units such as pediatrics, surgery, eye care, psychiatry, and pediatric care.

### Participants

The study targeted of 2980 licensed nurses employed in 13 government hospitals.

#### Inclusion criteria

All nurses should meet the following criteria: (1) nurses have at least a diploma degree; (2) employed in the 13 governmental hospitals (3) work experience of at least 6 months, (3) and fixed nurses.

#### Exclusion criteria

Newly appointed nursing staff, part-time nurses, and the nurses have administrative roles were excluded.

### Sample size and sampling method

The sample size for this study was estimated at 786 based on Krejci and Morgan’s table (margin of error = 3%, confidence level = 95%). In fact, when the total population under study is known, the simplest and easiest way to estimate the study’s sample size is the Morgan Table [15]. Hence, 786 male and female nurses in the surveys were proportionately selected based on the number of nurses in each hospital. A simple random sampling method was used to select participants from each hospital.

### Intervention

The conventional adult BLS training was carried out by BLS-qualified trainers in collaboration with the head of the in-service health education department at each hospital. PowerPoint, movies, and hands-on exercises on CPR mannequins were used. The training was done for two hours per week for one month for each group, Before the start of the training, the pre-test was conducted, and after the end of the training, the post-test was conducted, the participants continued to practice various BLS roles including heart compression, artificial respiration, the recovery position, airway management, foreign body airway obstruction, Administer first aid to an unconscious patient, and use of the Automated External Defibrillator (AED). Each participant practiced BLS stages for 5 min and at least of 20 min in each group after practicing on mannequins under the supervision of the trainers. Each subject is practiced independently and taught as a critical skill. The training was mainly based on role-playing and simulation, with all groups implementing the same training methods.

### Measures and data collection

Before the start of the simulation-based training program, a pre-test was distributed and collected by a qualified, trained nurse in each hospital, and every questionnaire with missing data was excluded. After completion of the simulation-based training program, a post-test was distributed and collected by a qualified, trained nurse at each hospital, and every questionnaire with missing data was excluded. The questionnaire included three variables related to the participant’s age, gender, and current work hospital. In addition, the BLS practice test contains of 10 multiple-choice questions and adheres to the AHA (2020) guidelines [16, 17]. The questionnaire includes items regarding compression-to-ventilation ratio, correct steps to operate an AED, pulse checking and correct locations, BLS steps used for adults, vital characteristics of first-rate CPR, recommended BLS sequence for the 2020 ILCOR guidelines, and signs of an obstruction of the airway. The overall score for this tool was from 0 to 10, and a higher score revealed more knowledge about BLS.

### Data analysis

SPSS software, version 24 was used for the statistical analysis. The characteristics of the nurses were described by using descriptive statistics such as mean, standard deviation, and percentages. In addition, weighted mean was calculated, calculating weighted mean involves multiplying each data point by its weight and summing those products. Then sum the weights for all data points. Finally, divide the weight*value products by the sum of the weights [18]. T-Test and One-way analysis of variance (ANOVA) were applied to determine differences in means among groups. All tests were conducted at the 0.05 level of statistical significance.

## Results

### Characteristics of participants

Of the 786 questionnaires distributed, 700 questionnaires were analyzed (response rate: 89.1%). The results of this study revealed that, more than half of the nurses (55.7%) were male, while (44.3%) were female. The majority of nurses (84.4%) are under 40 years of age. Almost a third of the participants were from Al-Shifa medical complex, while the least number of participants were from the Psychiatric Hospital and Al- Naser Eye Hospital (Table 1).

**Table 1:** Demographic characteristics of the nurses (n = 700)

Variable	Frequency	Percentage %
**Gender**		
Male	390	55.7
Female	310	44.3
**Age(years)**		
20-30years	273	39.0
31–40 years	318	45.4
41–50 years	81	11.6
51–60 years	28	4.0
**Current work hospital**		
Kamal Odwan	17	2.4
Al-Naser Eye Hospital	12	1.7
Al-Najar	35	5.0
Psychiatric	12	1.7
Beit-Hanon	24	3.4
Al-Shifa Complex	207	29.6
Naser Complex	115	16.4
Al-Elemirate	31	4.4
Indonesia	54	7.7
European Gaza	93	13.3
Al-Dora	21	3.0
AL-Rantisi	62	8.9
Turkish	17	2.4

### Knowledge of the study participants before and after the training

Table 2 displays the weighted mean scores of nurses to the BLS practice test questions according to the AHA (2020) guidelines. The weighted mean score in the pre-test ranged from 52.2 to 75.1%, while in the post-test they ranged from 85.6 to 97.3%. The nurses’ weighted mean score was higher on the post-test in the 10 questions included.

**Table 2:** Knowledge of the study participants before and after the training

Question	Pretest	Post-test
	Weighted mean (%)	Weighted mean (%)
What is the compression-to-ventilation ratio you should use when giving CPR to any individual?	52.2	93.1
When performing two-rescuer CPR, how often should you switch roles?	52.7	88.1
When operating an AED, what are the correct steps to follow?	61.4	85.6
When looking for a pulse on a child from one year to puberty, where should you check?	69.4	92.4
What are the BLS (Basic Life Support) steps used for adults?	52.3	93.6
What are the vital characteristics of first-rate CPR?	75.1	97.3
Which step is NOT a part of the five steps in the Adult Chain of Survival?	57.4	93.6
What is the recommended BLS sequence for the 2020 ILCOR guidelines?	66.6	94.4
What are the signs of an obstruction of the airway?	68.9	92.6
How many breaths should be given during a two-rescuer CPR on an adult with an advanced airway in place?	60.1	89.1
**Overall Weighted mean score (%)**	**61.6**	**91.9**

### Knowledge Mean scores before and after training

Table 3 displays that the mean knowledge was statistically significant between the pre and post-test. Nurses ' mean scores in the pre-test was (6.16 ± 1.97) and the mean score in the post-test was (9.19 ± 1.04).

**Table 3:** Knowledge mean score before and after training

	Mean	SD	P-value
**Pre-Test**	06.16	1.97	**< 0.001**
**Post-Test**	09.19	1.04	

SD: Standard Deviation. Differences between means were tested by using the Paired t-test.

### Knowledge mean scores before and after training based on gender

Table 4 displays that the mean scores of knowledge were non-statistically significant between the pre and post-test on the basis of gender. The mean scores for the pretest were (06.21 ± 1.96 and 06.18 ± 1.98) for males and females, respectively (p = 0.361). The mean scores for the pretest were (09.19 ± 1.06 and 09.20 ± 1.01) for males and females, respectively (p = 0.948).

**Table 4:** Knowledge mean scores before and after training based on gender

Gender (N)		Mean	SD	P- value
**Pre-Test**	Male (390)	06.21	1.96	0.361
	Female (310)	06.08	1.98	
**Post-Test**	Male (390)	09.19	1.06	0.948
	Female (310)	09.20	1.01	

Differences between means were tested by using the Independent-samples t-test

### Knowledge mean score before and after training based on age groups

Table 5 displays that the mean scores of knowledges were non-statistically significant between the pre and post-test on the basis of age group. The highest mean score in the pretest was registered in the age group 31–40 years old (06.24 ± 1.9) and the lowest mean score was registered in the age group 20–30 years old (06.06 ± 2.11). The highest mean score in the post-test was registered in the age group 51–60 years old (09.35 ± 0.78) and the lowest mean score was registered in the age group 31–40 years old (09.13 ± 1.04).

**Table 5:** Knowledge mean scores before and after training based on age groups

Age group/Years (N)		Mean	SD	P- value
**Pre-Test**	20–30(273)	06.06	02.11	0.725
	31–40 (318)	06.24	01.91	
	41–50 (81)	06.08	01.81	
	51–60 (28)	06.17	01.70	
**Post-Test**	20–30(273)	09.20	01.07	0.343
	31–40 (318)	09.13	01.04	
	41–50 (81)	09.34	00.98	
	51–60 (28)	09.35	00.78	

Differences between means were tested by using the One-Way ANOVA

### Knowledge mean scores before and after training based on the current work hospital

Table 6 shows that the mean scores of knowledges were statistically significant between the pre and post-test on the basis of the current work hospital (P-value < 0.001). In the pre-test, the highest mean score was registered in the Turkish Hospital located in Gaza governorate (08.35, SD ± 1.04). The lowest was in Kamal Odwan Hospital (04.64, SD ± 2.20) located in North-Gaza governorate. In the post-test, the highest mean score was registered in Al-Dora Hospital located in Gaza governorate (10.00, SD ± 0.20). The lowest was registered in Kamal Odwan Hospital (07.58, SD ± 1.32) located in North-Gaza governorate.

**Table 6:** Knowledge mean scores before and after training based on the current work hospital

Current work hospital	Pre-test			Post-test		
	Mean	SD	P-value	Mean	SD	P-value
Kamal Odwan (17)	04.64	2.20	**< 0.001**	07.58	1.32	**< 0.001**
Al-Shifa Complex (207)	06.20	2.0		09.14	1.09	
Naser Complex (115)	06.35	1.71		09.12	0.98	
Al-Elemirate (31)	06.25	1.73		09.51	0.72	
Indonesia (54)	06.24	2.17		09.11	0.94	
European Gaza (93)	05.94	2.12		09.65	0.56	
Al-Dora (21)	05.23	1.41		10.00	0.20	
AL-Rantisi (62)	05.45	1.87		09.25	0.95	
Turkish (17)	08.35	1.49		09.11	1.05	
Al- Naser Eye (12)	07.33	1.49		07.91	1.31	
Al-Najar (35)	06.57	1.37		09.31	0.75	
Psychiatric (12)	05.50	1.73		08.16	1.64	
Beit-Hanon (24)	06.58	2.06		09.29	1.04	

Differences between means were tested by using the One-Way ANOVA

## Discussion

A simulation training is a dynamic method used to correlate actual clinical conditions in a safe area that supports nurses to grow knowledge and psychomotor skills resuscitation [19]. To the best of our knowledge, there are no previous studies about BLS training on nurses’ knowledge in Palestine. Therefore, the study aimed to evaluate the effect of simulation-based BLS training on nurses’ knowledge among nurses at public hospitals in the Gaza strip, Palestine.

The results of this study showed that the weighted mean scores in the pre-test ranged from 52.2 to 75.1% and the mean score was (6.16 out of 10.0 ± 1.97). Although our participants had a mean score higher than the mean score in a study conducted among nurses in Turkey (5.66 ± 1.97) [20]. The current findings indicate the need for urgent intervention to enhance the knowledge of Palestinian nurses in BLS. It has been stated that BLS is a dynamic issue that needs continuous and simultaneous training. There is a relationship between BLS knowledge, nurse practice, and cardiopulmonary resuscitation (CPR) consequences [21]. After applying conventional BLS training for adults by qualified BLS instructors and participants practiced BLS steps in the Clinical Skills Center, the weighted mean scores in post-test they ranged from 85.6 to 97.3% and the mean score was (9.19). The study displayed that the nurses’ knowledge enhanced after implementing simulation-based training. These outcomes are consistent with earlier studies that showed a significant effect of CPR training on knowledge and practice [22–25]. The results of a study conducted among undergraduate nursing students in Thailand showed that the training had a significant and immediate impact on knowledge, self-efficacy, and chest compression skill [22]. Another study compared the knowledge and skills of undergraduate and staff nurses showed that the knowledge and skills of both groups of nurses improved with training [23].

The current study findings showed that the mean scores of knowledges were non-statistically significant between the pre and post-test on the basis of gender and age group. Similar results were found in a study carried out among nurses in Kuwait hospitals [26]. The mean scores of knowledges were statistically significant between the pre and post-test on the basis of the current work hospital (P-value < 0.001). In the pre-test, the highest mean score was registered in the Turkish Hospital located in Gaza governorate. The lowest was in Kamal Odwan Hospital located in North-Gaza governorate. In the post-test, the highest mean score was registered in Al-Dora Hospital located in Gaza governorate. The lowest was registered in Kamal Odwan Hospital located in North-Gaza governorate. The current findings can be explained by the fact that the skills of health care workers’ in some governorates of the Gaza Strip are poorly developed or underused, inequitable distribution of health care workers within the governorates of the Gaza Strip and the focus is on the main governorate (Gaza) [14, 27, 28]. Nursing managers can implement systematic strategies to enhance nurses’ knowledge and practice in BLS to target low-scoring Governorates.

### Implication for decision makers and management

The study findings can enhance insight and understanding of the effects of the BLS training program in Nurses’ knowledge. We suggest some practical implications for managers. Advanced BLS training for all healthcare providers, doctors, and nurses working in hospitals and healthcare centers. Training in BLS must be systematic and continuous to increase confidence in the use of CPR and potentially save lives. Then, it is needed to evaluate the impact and of these interventions. Future research should be expanded to include government hospitals in the West Bank, private institutions, non-governmental organizations and UNRWA.

### Strengths and limitations

The current study is one of the few studies that evaluates the effect of BLS training on nurses’ knowledge among nurses in government hospitals in the Gaza Strip, Palestine. The study provides important evidence of the positive effects of the BLS training program in improving nurses’ knowledge. This study has several limitations including the use of a quasi-experimental design without a control group. The duration of the program was short, and it needed to enrich the educational program and improve the simulation laboratories in the Gaza Strip. In addition, no follow-up was conducted to measure the impact of the program on patient safety. Moreover, there are other limitations, which are that the study included hospitals in the Gaza Strip only and did not include hospitals in the West Bank. It was conducted in government hospitals and did not include private institutions, non-governmental organizations and UNRWA.

## Conclusion

This study provides significant evidence of the positive effects of the BLS training program in improving nurses’ knowledge; we recommend advanced BLS training for all healthcare providers, doctors, and nurses working in hospitals and healthcare centers. Training in BLS must be systematic and continuous to increase confidence in the use of CPR and potentially save lives. Healthcare professionals must work together to ensure that these courses are successfully received and completed. Future research should target healthcare providers in emergency departments who are the first possible contact with patients who require CPR. In addition, nursing managers can implement systematic strategies to enhance nurses’ knowledge in BLS to target low-scoring Governorates.

## Data Availability

The datasets used and/or analyzed during the current study are available from the corresponding author upon reasonable request.

## List of abbreviations

BLS: Basic Life Support
AHA: American Heart Association
CPR: Cardio Pulmonary Resuscitation
AED: Automated External Defibrillator
